# Prevalence of modifiable cardiovascular risk factors in Yazd inner-city municipalities

**DOI:** 10.1186/s12889-020-8217-8

**Published:** 2020-01-30

**Authors:** Mohsen Mirzaei, Masoud Mirzaei, Ali Reza Sarsangi, Nasser Bagheri

**Affiliations:** 1 Yazd Cardiovascular Research Centre, Shahid Sadoughi University of Medical Sciences, Yazd, Iran; 2 Yazd Cardiovascular Research Centre, Shahid Sadoughi University of Medical Sciences, Jomhuri Blvd. Afshar hospital, Yazd, Iran; 3 Department of Remote Sensing and GIS, Faculty of Geography, University of Tehran, Tehran, Iran; 4 Visualization and Decision Analytics (VIDEA) lab, Centre for Mental Health Research, Research School of Population Health, College of Health and Medicine, The Australian National University, Canberra, Australia

**Keywords:** Cardiovascular risk factors, Municipality, Yazd, Urban, Prevalence

## Abstract

**Background:**

Cardiovascular disease (CVD) is the leading cause of death in the world. With effective intervention and control of cardiovascular risk factors, mortality rates may be reduced. The aim of this study was to investigate the prevalence of modifiable risk factors across five municipalities in Yazd city.

**Methods:**

Ten thousand residents of the Yazd greater area aged 20–69 years were selected using cluster random sampling method. Overall, 200 clusters were randomly selected based on the postcodes of residents who lived in the five municipalities of Yazd. Those who lived in Yazd annexed cities and rural areas were excluded. A valid questionnaire was completed and physical examination performed as done (94.9% response rate). Instances of self-reported diabetes mellitus, high blood cholesterol, tobacco smoking, and unhealthy diet were recorded. Blood pressure, height, and weight were measured and physical activity was classified by International Physical Activity *Questionnaire* (IPAQ). A chi-square test was used to analyze the differences in variables across municipalities. Statistical analyses were performed using SPSS V. 16.

**Results:**

We analyzed 8749 participants’ data from Yazd city. The prevalence of diabetes mellitus, hypercholesterolemia, and hypertension were 14.1, 16.7 and 18.6%, respectively. One in every four people consumed the recommended five servings of vegetables per day. Fish consumption was less than 5% at least once a week among participants. An unhealthy diet (85.7%); low physical activity (52.2%), hypertension (36.7%) and obesity (26.3%) were the most common cardiovascular risk factors. Only 2.1% of adults had no risk factors for CVD, and almost 75% of people had more than one risk factor. The prevalence of risk factors (excluding hypertension) was significantly different across the municipalities. Residents of region three had the highest prevalence of all risk factors aside from inactivity and unhealthy diet.

**Conclusion:**

unhealthy dietary habits and inactivity are the most common modifiable risk factors of CVD in Yazd. Spatial variations of cardiovascular risk factors observed. This geographic health inequality requires more attention from policymakers to control CVD risk factors across different municipalities accordingly. Promoting healthy lifestyle is the top priority of health intervention programs. It is recommended to increase access to sport arenas and restrict access to tobacconist in high-risk areas.

## Background

Cardiovascular disease (CVD) is the leading cause of death in the world [1]. By 2030, 23 million people are predicted to die annually due to CVD, and deaths will be mainly clustered in low-middle income countries [2]. Because of the increasing rates of mortality due to Non-Communicable Diseases (NCDs), especially in developing countries, the World Health Organization (WHO) has listed these diseases as a health priority and has proposed nine global targets for preventing and controlling these diseases. They aim to reach the goal of a 25% reduction by 2025 in premature deaths from cardiovascular disease, cancer, diabetes, and chronic respiratory diseases [3, 4].

Cardiovascular disease accounts for about 50% of reported deaths in Iran [5, 6]. Ischemic heart diseases and stroke are the most common cause of death in Iran. The burden of CVD is increasing in Iran. CVD death rates increased between 2007 and 2017 in Iran; ischemic heart disease by 27.4% and stroke by 19.6% [7]. Due to an increased level of life expectancy over the past few decades in Iran, the population is aging and the country is experiencing a demographic transition. Thus, the diseases and causes of death have changed, in line with epidemiological transition, and this trend is expected to continue [5].

More than 75% of early-onset CVDs can be prevented and intervention to control risk factors can help reduce cardiovascular burden [8]. Cardiovascular risk factors such as tobacco and alcohol use, inadequate physical activity, an unhealthy diet can be altered by lifestyle changes. If CVD’s risk factors diagnosed early and managed with effective prevention and treatment interventions, this would help to control and reduce the associated CVD morbidity and mortality [9–11].

In Iran, an unhealthy diet and metabolic risk factors are the most common risk factors for NCDs followed by tobacco smoking, air pollution, low physical activity, and alcohol consumption [4]. The association between cardiovascular disease and their risk factors and socioeconomic factors has been reported in many studies [12–14]. A relationship between social determinants and lifestyle behaviors like physical inactivity, tobacco smoking, and dietary habits and cardiovascular disease mortality has been reported [15, 16] as well as the relationship between Socio-Economic Status (SES) and Type-2 diabetes mellitus(T2DM) [17], hypercholesterolemia [18], obesity and high blood pressure [19] has been documented. The historical city of Yazd has a new urban texture along with the old area. Because of immigration, In Yazd people live in different cultures and lifestyles. The development of industries and the horizontal expansion of the city have led the regions to expose by different health risk factors. Identifying cardiovascular risk factors in each area can help for effective interventions to modify unhealthy lifestyle.

The aim of this study was to investigate modifiable cardiovascular risk factors related to lifestyle including; dietary habits, tobacco smoking and physical inactivity of Yazd residents. Metabolic risk factors including obesity, diabetes mellitus, high blood cholesterol, and hypertension were estimated in each region, which answers the question of whether the prevalence of these risk factors varies across the different municipalities of Yazd city. The findings helps health policymakers in regional health planning.

## Methods

### Setting and study design, data collection

Yazd Health Study (YaHS) is a prospective cohort study conducted to determine the prevalence of non-communicable disease and related risk factors in the Yazd Greater Area (YGA), which is located in central Iran. The present study is a cross-sectional analysis of the Yazd Health Study (YaHS) recruitment phase data of YaHS is a prospective cohort study.

### Study area

**Fig. 1:**
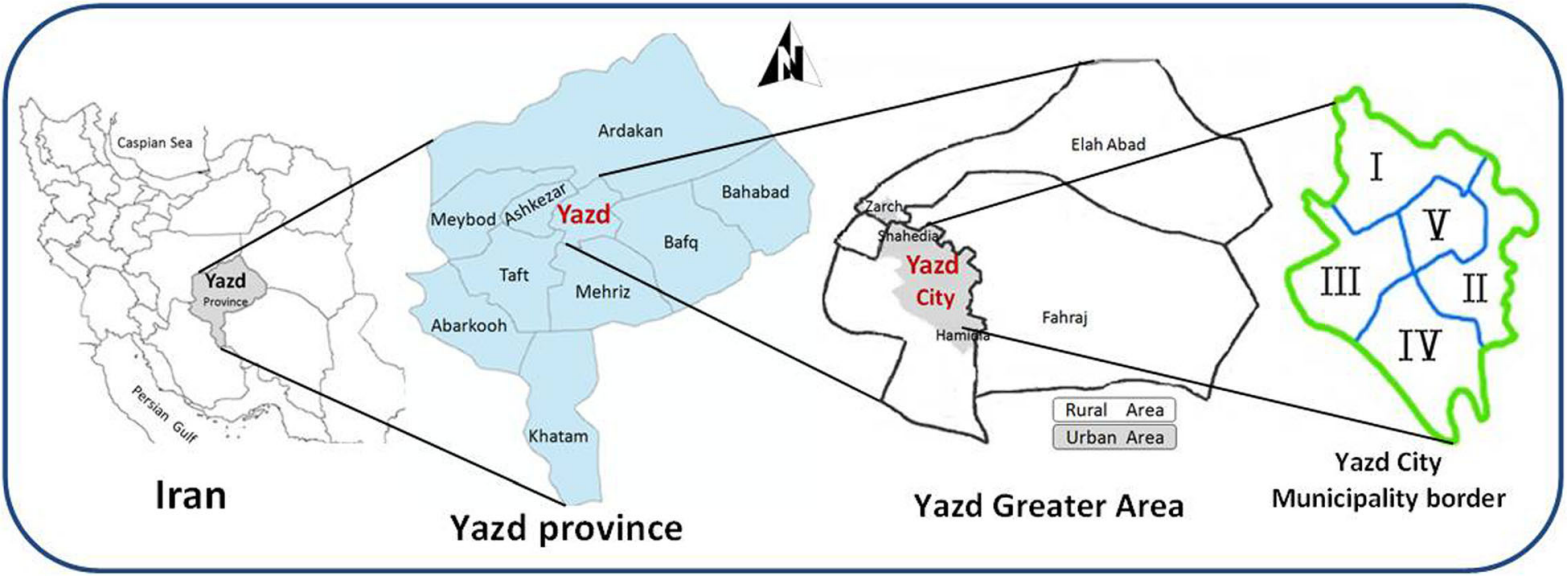
The map of Yazd city, including borders of the five municipalities.It was developed by the authors of the study

The historical city of Yazd is the capital of Yazd province (Iran) which was recognized as a World Heritage city by UNESCO in 2017. Yazd city is divided into 4 municipal districts and a special historical district (Fig. 1). Historical region (Yazd 5) and villages annexed to Yazd city accommodate a high proportion of immigrant residents, who are in lower socioeconomic class. These differences in social factors can affect the incidence and severity of heart disease risk factors.

### Sample size

Maximize sample size was calculated with prevalence of 50% and significance level 99%, for all the NCD and their risk factors. The initial sample size was calculated 538. It was corrected based on ten strata (20–29, 30–39, 40–49, 50–59, 60–69 by sex). We considered the design effect of 1.5, 5% non-response in recruitment phase (*n* = 8494) and 15% attrition rate or follow-up loss (*n* = 9768) in second wave. So, we decided to enroll 10,000 persons.

Ten thousand residents of YGA aged 20 to 69 years were selected using cluster random sampling method. 200 clusters were randomly selected based on participants’ residential postcode. Then, each cluster of 50 samples was divided into the following subgroups: 25 men and 25 women; five people in each sex in each ten-year age range categories (20–29, 30–39, 40–49, 50–59 and 60–69 years old). Trained interviewers completed a validated questionnaire and performed a physical examination (94.9% response rate). Sampling procedures and other details of the YaHS study have been published elsewhere [20]. Residents of Yazd annexed cities and rural areas were excluded from the analysis.Thus, 8749 individuals, who lived in five municipalities of Yazd city, were included in the final analysis.

To assess individual-level socioeconomic factors, self-reported information on education level, job status, health insurance, marital status, and migration status were recorded. The included cardiovascular risk factors were: T2DM (defined as history of diabetes mellitus and/or insulin drug therapy); dyslipidemia as defined by history and/or drug therapy for hypercholesterolemia; hypertension as defined by history of hypertension, a systolic blood pressure ≥ 140 mmHg and/or a diastolic blood pressure ≥ 90 mmHg and/or antihypertensive therapy [21] and d) overweight as defined by a body mass index (BMI) between 25 to 29.9 kg/m^2^ and obesity (BMI of ≥30 kg/m2) [22]. Lifestyle cardiovascular risk factors were also analyzed, including current tobacco and/or hookah smoking and a selected unhealthy diet (Eating < 2 servings of fruits/day, Eating < 2 servings of vegetable/day, Eating > 4 servings of red meat/week, Eating < 1 serving of fish /week [23] and low physical activity according to IPAQ short form (SF) questionnaire. The IPAQ-SF examines the intensity of physical activity last week for different levels. Weekly walking time, moderate-intensity and intense activity severity were calculated individually. Estimate metabolic equivalent (MET; multiples of resting energy expenditure) by minutes reported per week in each category. Finally, participants were classified into three levels of “low”, “moderate” and “high” physical activity [24, 25].

### Statistical analysis

Yazd population in 2011 was obtained from the Center of Statistics of Iran for direct age-standardization of the findings. Categorical variables are presented as frequencies/percentages and the prevalence of risk factors is described as proportions. A chi-square test was used for categorical variables to analyze the differences in variables between regions. All statistical analyses were performed using SPSS version 16 software. A *p*-value of less than 0.05 was considered statistically significant.

All procedures performed in this study were approved by the ethics committee of Shahid Sadoughi University of Medical Science (IR.SSU.MEDICINE.1396.311) and informed consent was obtained from all participants.

## Results

Of the total 8749 participants, 1722 (19.7%) lived in municipality region 1, 2099 (24%) lived in region 2, 1194 people (13.6%) lived in region 3, 2226 (25.4%) lived in region 4 and 1508 (17.2%) lived in the historic district.

13.3% of the residents were not native and migrated to Yazd from other provinces and 201 individuals (2.3%) from other countries. Of the included participants, 25.9% were illiterate or had elementary school-level education, 5.7% of them did not have insurance coverage and 68% of men were employed whereas 76.9% of women were housewives. Details of socio-demographic factors are presented in Table 1.

**Table 1:** Socio-economic and cardiovascular risk profile of participants

	Total *n* = 8749	Men *n* = 4349 (49.7%)	Women *n* = 4400 (50.3%)	*p* value
Native	7305 (86.7%)	3668(87.6%)	3637(85.7%)	0.007
Historical region residents	1508(17.2%)	756(17.4%)	752(17.1%)	NS
Married	7366(84.5%)	3643(84.1%)	3723(84.9%)	NS
High school or higher level of education	3993(46.1%)	2200(51.1%)	1793(41.1%)	< 0.001
Health insured	8126(94.3%)	4007(93.1%)	4119(95.4%)	< 0.001
Employed	3399(39.5%)	2907(67.9%)	492(11.4%)	< 0.001
Hypercholesterolemia	1446(16.7%)	579(13.4%)	867(19.9%)	< 0.001
Diabetes^a^	1224(14.1%)	531(12.3%)	693(15.8%)	< 0.001
Hypertension^a^	1601(18.6%)	652(15.3%)	949(21.9%)	< 0.001
Hypertension(measured)	2561(29.6%)	1438(33.5%)	1123(25.8%)	< 0.001
Obesity (Body mass index ≥30 kg/m2)	2273(26.3%)	787(18.3%)	1486(34.1%)	< 0.001
Current cigarette Smoking	958(11.3%)	880(20.7%)	78(1.8%)	< 0.001
Current water-pipe Smoking	759(8.9%)	590(13.9%)	169(4%)	< 0.001
Low physical activity	4567(52.2%)	2014(46.3%)	2553(58%)	< 0.001
Eating < 2 servings of fruits/day	4613(53.0%)	2287(52.9%)	2326(53.1%)	NS
Eating < 2 servings of vegetable/day	6713(77.2%)	3363(77.8%)	3350(76.5%)	NS
Eating < 1 serving of fish /week	8300(95.4%)	4103(95.2%)	4197(95.7%)	NS
Eating > 4 servings of red meat/week	2023(23.2%)	955(22.0%)	1068(24.3%)	0.006

^a^Self-reported

The prevalence of T2DM, dyslipidemia and high blood pressure in Yazd were 14.1, 16.7, and 18.6%, respectively. Findings also show that 11% of adults were not aware of their hypertension. Less than half of the residents consume two or more servings of fruit per day, and only one in every four people consumes the recommended five servings of fruits and vegetables per day. Less than 5% of the population ate fish at least once a week. (Table 1).

Table 2 shows the variation of socio-economic factors across municipalities. Municipality No.3 with 18.8% had the highest percentage of immigrants from other provinces and the historical region with 6.1% had the highest percentage of overseas immigrants (*p*-value < 0.0001). Municipality No.3 has the highest percentage of singles (13%); employed (43.9%) and health insurance coverage (96%) across the municipalities. The literacy level of the residents of this region is significantly higher than the other regions (Table 2).

**Table 2:** Proportion of participants with risk factors according to municipality

	Total	Yazd municipality No (%)					*p* value
		Yazd 1	Yazd 2	Yazd 3	Yazd 4	Yazd 5 (Historical)	
Socio-economic status							
Native	7305(86.7)	1459(86.4)	1771(85.6)	957(81.2)	1894(85.7)	1224(82.5)	< 0.001
Married	7366(84.5)	1424(83.4)	1796(85.7)	980(82.6)	1924(86.7)	1242(82.6)	0.006
High school or higher level of education	3993(46.1)	742(43.7)	913(43.8)	772(65.5)	908(41.1)	658(44.0)	< 0.001
Insured	8126(94.3)	1607(94.4)	1951(94.6)	1135(96)	2090(95.0)	1343(91.2)	< 0.001
Employed	3399(39.5)	653(38.5)	776(37.6)	512(43.9)	853(39.0)	605(40.7)	< 0.001
Modifiable Risk factor							
Diabetes	1224(14.1)	249(14.6)	311(14.9)	179(15.1)	266(12.0)	219(14.6)	0.03
Hypercholesterolemia	1446(16.7)	318(18.8)	330(15.9)	221(18.8)	337(15.2)	240(16.1)	0.007
Hypertension^a^	3214(36.7)	609(35.4)	789(37.6)	450(37.7)	834(37.5)	532(35.3)	NS
Obesity	2273(26.3)	465(27.2)	552(26.6)	339(28.6)	530(24.1)	387(26.1)	0.049
Tobacco Smoking^b^	1596(18.5)	305(17.8)	396(19.2)	243(20.4)	356(16.0)	296(20.3)	0.003
Low physical activity	4567(52.2)	734(42.6)	1352(64.4)	545(45.6)	1262(56.7)	674(44.7)	< 0.001
Unhealthy diet^c^	7386(85.7)	1506(89.2)	1784(86.6)	933(79.2)	1874(85.1)	1289(86.7)	< 0.001
Number of risk factors							
0	178(2.1)	40(2.5)	23(1.2)	36(3.2)	42(2.0)	37(2.7)	< 0.001
1	1725(20.8)	398(2.1)	337(2.5)	249(1.2)	444(3.2)	297(2.0)	
2	2677(32.3)	479(29.4)	647(32.6)	346(30.4)	730(34.0)	475(34.1)	
3	1986(23.9)	368(22.6)	514(25.9)	274(24.0)	521(24.2)	309(22.2)	
4	1091(13.2)	209(12.8)	288(14.5)	157(13.8)	262(12.2)	175(12.6)	
≥ 5	639(7.7)	135(8.3)	175(8.8)	78(6.8)	150(7.0)	101(7.2)	

^a^ History or measurement

^b^ Cigarette or water-pipe smoking

^c^ Inappropriate fruit, vegetable, red meat and fish consumption (3 of 4)

An unhealthy diet, low physical activity, hypertension, and obesity were the most common cardiovascular risk factors, occurring at a frequency of 85.7, 52.2, 36.7, and 26.3% respectively. There is a significant difference between the prevalence of risk factors among municipalities, except prevalence of hypertension which is not statistically significant.

The highest prevalence of T2DM, hypercholesterolemia, tobacco smoking, and obesity was observed in the residents of municipality No.3. Further, low physical activity in municipality No.2 and unhealthy diet in No. 1 are more prevalent among the regions. (Table 2).

**Fig. 2:**
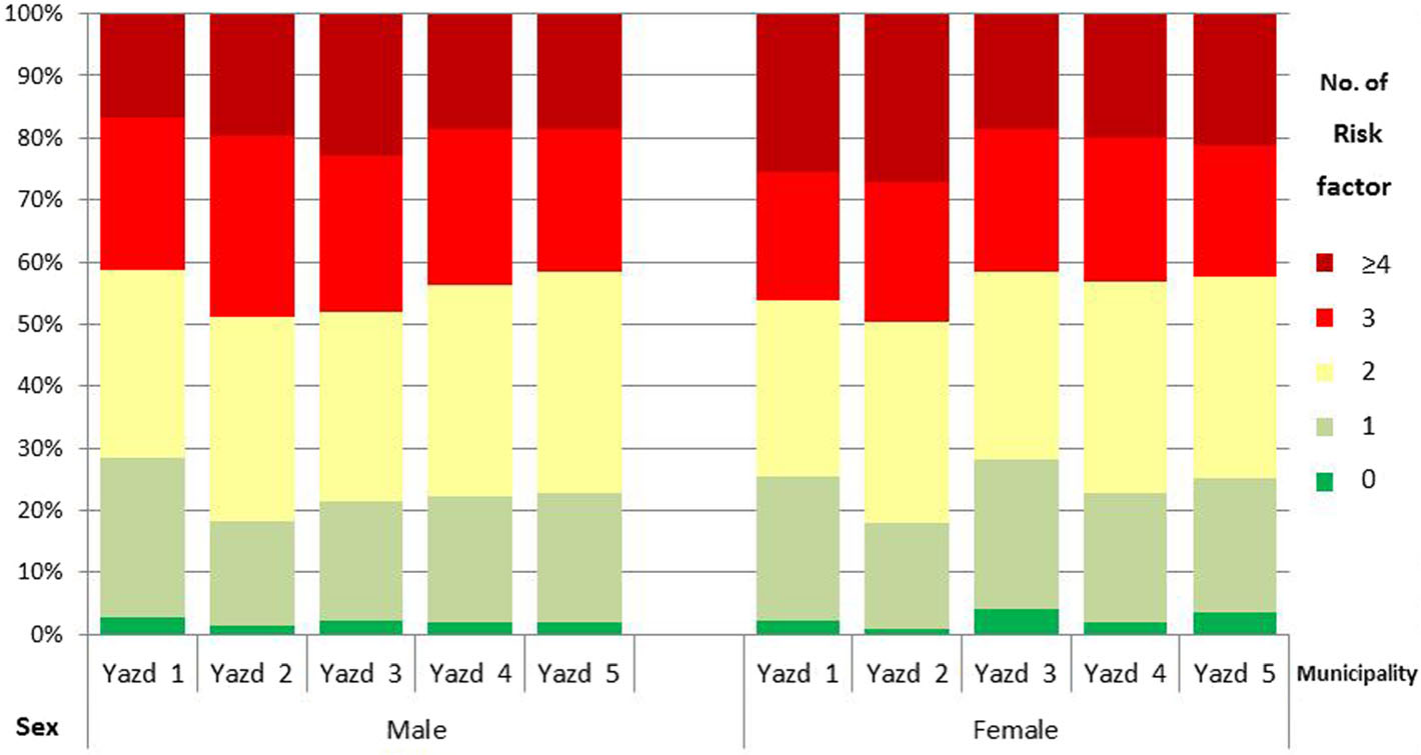
Number of cardiovascular risk factors in participants by sex and municipality of residence in Yazd city

Only 2.1% of the adults had no risk factors for CVD, and over 75% had more than one risk factor. Table 2 shows the difference in the prevalence of risk factors across municipalities. (Fig. 2).

## Discussion

The prevalence of cardiovascular risk factors in Yazd is high and notable. This study shows significant regional variations in CVD risk factors prevalence in Yazd city. An unhealthy diet, low physical activity, and high blood pressure are the three most common risk factors in Yazd city. Obesity and tobacco smoking were ranked second. The prevalence of risk factors is similar in men and women except that in males, tobacco smoking is ranked higher than obesity. Low fish and vegetable consumption are the most important unhealthy dietary behaviors in Yazd city.

### Unhealthy dietary behavior

In the population of Yazd, 95.4% of adults consume fish less than once a week and 23.2% eat red meat more than four times a week. Fruit and vegetable intake (less than twice a day) are seen in 53.2 and 77% of the individuals, respectively. An unhealthy eating pattern was present in 85.7% of Yazdi adults with a significant difference of 10% between urban and rural areas, indicating the impact of socioeconomic factors on health behaviors. The residents of region 3 had the highest percentage of fish, vegetables and fruit consumption compared to the other regions in the study area. Food habits influenced by a set of variables including food availability, preparation, socioeconomic status, local culture, religion, and media advertisement [26]. Health policymakers need to take into account these relationships in policy planning and interventions.

### Hypercholesterolemia

The prevalence of hypercholesterolemia in Yazd (16.7%) is lower than other studies in Iran (41.6%), Saudi Arabia (54%), Turkey (37.5%), and Europe (56.7%) and even less than the previous study in Yazd itself (35.4%), although consumption varies across Yazd regions [27, 28].

Reducing red meat consumption can be effective in decreasing the risk of CVD. Educational interventions; Physicians ‘and health care providers’ recommendations are one of the causes of changes in the consumption of fatty foods and the low prevalence of cholesterol in the community. Family income and food price changes, including red meat, can also contribute to household consumption.

### Obesity

The prevalence of obesity in this study is higher than the findings of other national and local studies in Iran, especially in women (almost twice as high) [29]. Although the observed difference in obesity between residents of various Yazd municipalities is not remarkable, these differences were reported by other studies too [30, 31]. Considering the relationship between obesity and dietary pattern and physical inactivity, and the difference between these two behaviors among residents of Yazd municipalities, as seen in this study, this difference in obesity prevalence between areas was expected.

### Physical inactivity

Physical activity is not enough in half of the adult population of Yazd with a 20% difference across Yazd municipalities (e.g. 42.6% in municipality 1 vs. 64.4% in municipality 2). Inadequate physical activity in some areas is remarkably lower than the country average [32]. Physical inactivity in Yazd is more common than Kerman and the national average, although the findings of a previous study in Tehran and Yazd show a lower level. These differences may be due to different measurement tools. The apparent relationship between lower physical activity in women and lower SES aligns with results similar to other, similar studies [28, 33, 34]. Cultural constraints and the lack of adequate indoor and outdoor sports facilities space can also affect people’s physical activity. The lack of attention to the green-sports spaces and the service distance in Yazd urban development projects has caused limited access in some municipalities, especially 1–2 and the marginal area [35]. Also, more expensive spending in the private sector can limit to access for deprived areas. Inequality in the availability of public or private sporting equipment and the distance to sporting venues may affect this difference [36].

### Tobacco smoking

The study shows that 18.5% of adults engage in tobacco use (cigarette or water-pipe), which is lower than the reported national prevalence rates. Similar to other studies conducted in Iran, men smoke more than women, although the gap is smaller for water-pipe usage [37]. An investigation of female tobacco consumption has also shown an alarming upward trend in recent years, in Yazd [38]. Significant differences in the prevalence of smoking in urban areas may show the role of social determinants on smoking, which has also been reported in other studies [38]. The geographic distribution of tobacconists, especially hookahs, in different parts of the city and the association of easy access to tobacco use can help in identifying the factors that influence usage.

### Diabetes mellitus

Physical inactivity, unhealthy diet, and obesity in urban areas are the most important risk factors of T2DM, which is a major CVD risk factor itself [39]. The differences in the prevalence of T2DM among regions may be reflective of socioeconomic level, and the lifestyle and dietary behavior associated with it, as explained above [40]. The results of our study indicated differences in geoghrafical patterns of T2DM, as reported in other studies [41]. A further study of the contribution of each dimension of socioeconomic level and their differences across regions is necessary to allow effective preventive interventions for these non-communicable diseases. The higher prevalence of T2DM in Yazd compared to other provinces, as well as its increasing trend compared to previous years [28], requires a further ethnic and geographical investigation of the prevalence of T2DM and its risk factors.

### Hypertension

The prevalence of hypertension in Yazd is also higher than the country average and is occurring at a higher rate compared with previous years [28]. Obesity, tobacco smoking, sedentary lifestyle, unhealthy diet, and economic status are known risk factors for high blood pressure. Although this study investigated the differences in prevalence, of these risk factors across urban areas, unlike other studies [42, 43], the prevalence of high blood pressure across Yazd municipalities was not statistically significant. Given the history and physical examination of the participants, the high prevalence of hypertension in all areas of Yazd is alarming.

### Strength and weakness

This study, for the first time, addresses the differences in the risk factors of NCDs diseases in different regions of Yazd. Multi-stage random sampling using a standard protocol from all neighborhoods in Yazd, with 95% of respondents participating, is one of the strengths of this study. A high sample size with sufficient power provides an estimate of regional prevalence. Although this descriptive study cannot determine the cause of the most common risk factors for cardiovascular disease in a region compared to other areas. Alcohol consumption is a known risk; however this study has not investigated it.

## Conclusion

Each risk factor had a different prevalence/distribution among municipalities, suggesting the potential role of regional factors. Although people living in different areas belonging to different ethnicities and cultures, this finding may also be explained by the influences of varying environmental and socio-economic factors. The higher percentage of people had two or more risk factors for cardiovascular disease in Yazd (77%) and a significant difference between different urban areas is alarming. This geographic health inequality requires more attention by health policymakers to tailor the targeted intervention (e.g. SES intervention) to the right people at the right place. It also helps policymakers to effectively allocate the limited resources to the communities that are most in need. Community-based public awareness about having a healthy diet pattern, increasing physical activity level, and weight control, and limiting access to fast food and cigarette sales are among the appropriate interventions to decreases the risk of developing NCDs in high-risk areas. Further geospatial analyses of NCDs risk factors at small area levels are suggested.

## Data Availability

The data collected by Yazd Health Study are not open access but can be shared under conditions of collaboration and endowment. Data are available from the authors upon reasonable request and with permission of principal investigator. For further information, please visit YaHS website at www.yahs-ziba.com / www.yahs.ir.

## Abbreviations

CVD: Cardiovascular Disease
IPAQ-SF: International Physical Activity *Questionnaire*-Short Form
NCD: Non-Communicable Diseases
SES: Socioeconomic Status
T2DM: Type 2 Diabetes Mellitus
WHO: World Health Organization
YaHS: Yazd Health Study
YGA: Yazd Greater Area
